# Defining the causes for Fontan circulatory failure in total cavopulmonary connection patients

**DOI:** 10.1093/icvts/ivae188

**Published:** 2024-11-20

**Authors:** Joeri Van Puyvelde, Filip Rega, Werner Budts, Alexander Van De Bruaene, Bjorn Cools, Marc Gewillig, Benedicte Eyskens, Ruth Heying, Thomas Salaets, Bart Meyns

**Affiliations:** Department of Cardiac Surgery, University Hospitals Leuven, Leuven, Belgium; Department of Cardiovascular Sciences, KU Leuven, Leuven, Belgium; Department of Cardiac Surgery, University Hospitals Leuven, Leuven, Belgium; Department of Cardiovascular Sciences, KU Leuven, Leuven, Belgium; Department of Cardiovascular Sciences, KU Leuven, Leuven, Belgium; Department of Cardiology, University Hospitals Leuven, Leuven, Belgium; Department of Cardiovascular Sciences, KU Leuven, Leuven, Belgium; Department of Cardiology, University Hospitals Leuven, Leuven, Belgium; Department of Cardiovascular Sciences, KU Leuven, Leuven, Belgium; Department of Paediatric Cardiology, University Hospitals Leuven, Leuven, Belgium; Department of Cardiovascular Sciences, KU Leuven, Leuven, Belgium; Department of Paediatric Cardiology, University Hospitals Leuven, Leuven, Belgium; Department of Cardiovascular Sciences, KU Leuven, Leuven, Belgium; Department of Paediatric Cardiology, University Hospitals Leuven, Leuven, Belgium; Department of Cardiovascular Sciences, KU Leuven, Leuven, Belgium; Department of Paediatric Cardiology, University Hospitals Leuven, Leuven, Belgium; Department of Cardiovascular Sciences, KU Leuven, Leuven, Belgium; Department of Paediatric Cardiology, University Hospitals Leuven, Leuven, Belgium; Department of Cardiac Surgery, University Hospitals Leuven, Leuven, Belgium; Department of Cardiovascular Sciences, KU Leuven, Leuven, Belgium

**Keywords:** Fontan, Restrictive pathophysiology, Fontan failure, Univentricular heart, systolic ventricular dysfunction

## Abstract

**OBJECTIVES:**

This study aims to identify the causes of failure in Fontan patients with a total cavopulmonary connection.

**METHODS:**

We conducted a comprehensive review of all patients who underwent a total cavopulmonary connection procedure at our centre between 1988 and 2023, aiming to identify and analyse the factors contributing to Fontan failure (defined as mortality, heart transplantation, Fontan takedown, protein-losing enteropathy, plastic bronchitis or New York Heart Association Functional Classification class III or IV).

**RESULTS:**

The study included 217 patients (median age at time of Fontan completion 3.7 years) with a median follow-up of 12.7 years (interquartile range 7.2–17.7). Systolic ventricular function decreased significantly over time in patients with right ventricular dominant morphology (*P* = 0.002), while systolic ventricular function remained stable in patients with left ventricular dominant morphology. Fontan failure occurred in 24 patients, with estimated freedom from Fontan failure rates of 97.7% [95% confidence interval (CI), 95–99] at 1 year, 93.9% (95% CI, 89–97) at 15 years and 77.2% (95% CI, 65–86) at 20 years of follow-up. Systolic ventricular dysfunction was the most common cause of failure (29%), followed by atrioventricular valve regurgitation (16.7%), a high pulmonary vascular resistance (16.7%), restrictive pathophysiology (16.7%) and obstruction (12.5%). Patients with right ventricular dominance developed most often systolic ventricular dysfunction, while patients with left ventricular dominant morphology developed most often restrictive pathophysiology or a high pulmonary vascular resistance.

**CONCLUSIONS:**

Approximately 10% of patients experienced Fontan failure within 15 years postoperatively. Patients with right ventricular dominance experienced progressive decline due to systolic dysfunction, while those with left ventricular dominance exhibited failure due to restrictive pathophysiology or high pulmonary vascular resistance.

## Graphical Abstract

**Figure:**
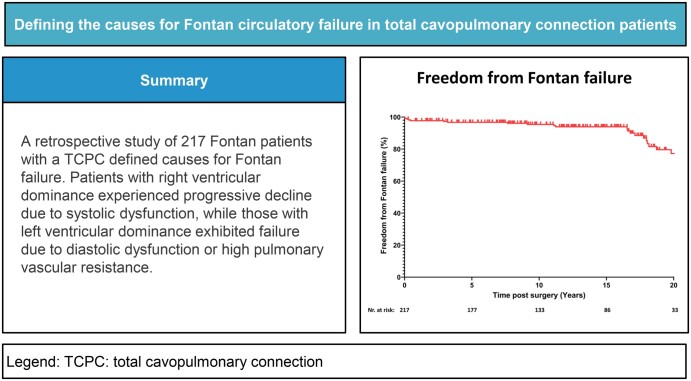

## INTRODUCTION

The Fontan procedure has revolutionized the palliative treatment of patients with a functional single ventricle [1]. Consecutive innovations in surgical techniques and postoperative management have greatly improved outcome, with a reported 95% survival at 10 years postoperatively for those patients with an extracardiac conduit total cavopulmonary connection (TCPC) [2, 3]. Despite the improvements in outcomes, Fontan patients still face significant challenges. Approximately one-third of adult Fontan patients are estimated to be living with a failing Fontan circulation [2, 4], necessitating a deeper understanding of the underlying mechanisms of Fontan failure. ‘Fontan failure’ is a variably defined term used to describe those patients with severe complications resulting in clinical deterioration. This term encompasses a heterogeneous group of patients with distinct underlying mechanisms of failure, including structural lesions of the Fontan pathway (e.g. conduit or pulmonary artery stenosis), atrioventricular valve (AVV) regurgitation, systolic ventricular dysfunction, high pulmonary vascular resistance (PVR) and the development of restrictive pathophysiology [5–10].

Defining the different causes of failure in Fontan patients who have undergone TCPC is essential for guiding individualized treatment plans. This retrospective study aims to analyse a group of patients who have undergone TCPC to shed light on the various causes of failure and provide insights into improving the management of this complex patient population.

## PATIENTS AND METHODS

### Ethics statement

Study approved by Institutional Review Board (UZ/KU Leuven Ethics Committee; S68932; 30/04/2024). Individual consent waived due to retrospective design and anonymity.

### Participant selection

This study included all patients who underwent Fontan completion with an extracardiac or intracardiac conduit TCPC at the University Hospital Leuven, Belgium, between November 1988 and May 2023. The patients were identified through a search of the surgical database, and data were collected through retrospective chart review.

### Institutional policy regarding Fontan strategy

Our surgical management for Fontan procedures has evolved over time. Currently (Era II: 1996–2024), we perform bidirectional cavopulmonary anastomosis at 6 months, followed by the Fontan procedure at 3 years or 15 kg. We use 18–20 mm extracardiac conduits with 4.5 mm fenestration and postoperative aspirin therapy for all patients. This approach differs from our earlier practice (Era I: 1988–1995), which involved single-stage procedures for older patients, the use of intracardiac conduits, and no routine fenestration. Due to these significant differences in patient characteristics and surgical techniques, we have stratified our patient cohort into these 2 eras.

### Definition and assessment of Fontan failure

**Figure 1:**
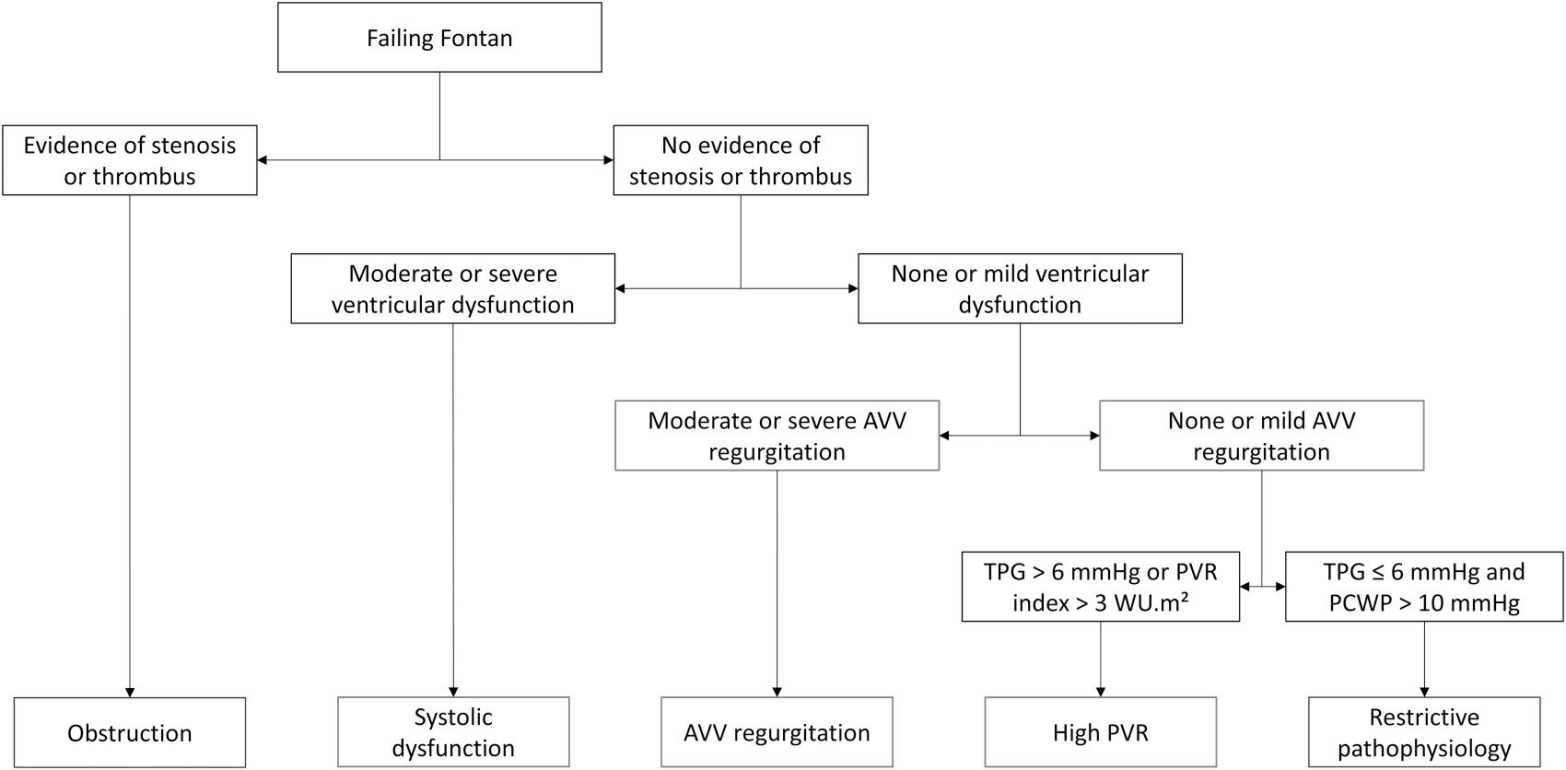
Flowchart for identifying the causes of Fontan failure. As multiple causes of failure can coincide and mutually influence each other, we prioritized the causes based on their assumed order of occurrence. AVV: atrioventricular valve; PCWP: pulmonary capillary wedge pressure; PVR: pulmonary vascular resistance; TPG: transpulmonary gradient.

Fontan failure was characterized as symptomatic insufficiency of TCPC circulation resulting in one or more of the following events: death, heart transplantation, Fontan takedown, protein-losing enteropathy (PLE) or plastic bronchitis, or New York Heart Association Functional Classification (NYHA) class III or IV at last follow-up. The underlying causes of failure were categorized as follows: Fontan obstruction, AVV regurgitation, systolic ventricular dysfunction, high PVR and restrictive pathophysiology. A flowchart (Fig. 1) was created to define and classify these causes of Fontan failure, prioritized by assumed occurrence order because they can coincide and mutually influence each other. Obstruction was defined as any indication of anatomical obstruction, such as stenosis or the presence of a thrombus. Systolic ventricular dysfunction was characterized as at least moderately reduced ventricular function. AVV regurgitation was categorized as moderate or severe. High PVR and restrictive pathophysiology were defined as a transpulmonary gradient (TPG) exceeding 6 mmHg or a PVR index exceeding 3 WU·m^2^ and as low TPG and ventricular filling pressures exceeding 10 mmHg, respectively [11].

Medical records of failing Fontan patients were reviewed, including echocardiography reports (ventricular function and AVV regurgitation severity) and cardiac catheterization data (TPG and signs of obstruction). Two investigators (J.V.P. and B.M.) independently assessed failure causes, reaching consensus on discordant cases.

### Data analysis

Continuous data are reported as median values with interquartile ranges (IQRs), while discrete data are presented as frequency (percentage). *P*-values < 0.05 were considered statistically significant, all *P* values being 2-sided. Clark’s Completeness Index was used to calculate the follow-up completeness. Complete case analyses were performed. A Friedman test was conducted to assess differences in ventricular function over time among patients with right ventricular (RV) and left ventricular (LV) dominance. Freedom from Fontan failure and transplant-free survival functions were generated by the Kaplan–Meier method. Additionally, we assessed the linearized risk of Fontan failure. All statistical analyses were performed using IBM SPSS statistics version 29 (IBM corp., Armonk, NJ, USA) and GraphPad Prism version 10.1.0 (Dotmatics, Boston, MA, USA).

## RESULTS

### Patient cohort

**Figure 2:**
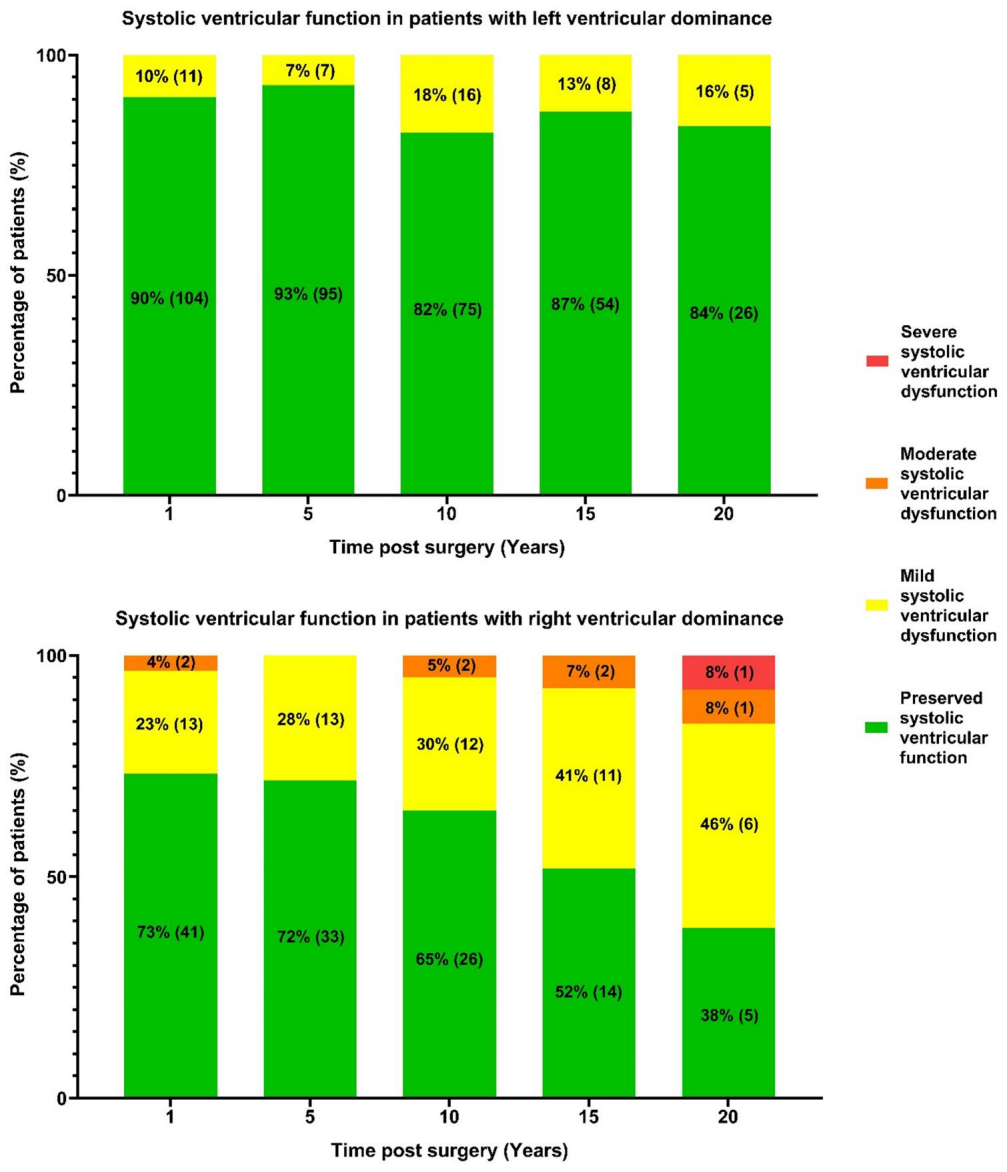
Systolic ventricular function over time in Fontan patients with left (top) and right (bottom) ventricular dominant morphology. Percentages of patients, with absolute numbers in parentheses, in 4 echocardiography-based function categories are presented. Right ventricular dominant morphology patients show increasing systolic dysfunction (*P* = 0.002), unlike left ventricular dominant morphology patients (*P* = 0.441).

Of 236 TCPC patients, 217 were included in the analysis (19 excluded due to Fontan conversion procedures). 18 patients were operated on during Era I (1988–1995) and 199 during Era II (1996–2024). Table 1 presents the demographic and clinical characteristics of the patient population. Most patients (60.8%) had a systemic LV. Tricuspid atresia accounted for a quarter of the patients, while 17% had a double inlet LV, 14% had a double outlet RV, and 10% were hypoplastic left heart patients. The median age at Fontan completion was 3.7 years (IQR, 3.2–4.7). A fenestration of the conduit was created during the TCPC operation in 190 patients (88.5%), and 28 (12.9%) still had an open fenestration at the last follow-up. The median follow-up duration was 12.7 years (IQR, 7.2–17.7) and completeness of follow-up for the cohort was 91.0%. In Fontan patients with RV dominance, RV function significantly decreased over time (*P* = 0.002), whereas in patients with LV dominance, LV function did not show a significant decrease during the follow-up period (*P* = 0.441) (Fig. 2).

**Table 1:** Patient demographics and pre-operative, operative, and postoperative characteristics of TCPC Fontan patients

		Era I (1988–1995)		Era II (1996–2023)	
	Fontan patients (*n* = 217)	No Fontan failure (*n* = 13)	Fontan failure (*n* = 5)	No Fontan failure (*n* = 180)	Fontan failure (*n* = 19)
Male	134 (61.8%)	8 (61.5%)	3 (60%)	111 (61.7%)	12 (63.2%)
Dextrocardia	17 (7.8%)	3 (23.1%)	2 (40%)	11 (6.1%)	1 (5.3%)
Isomerism	18 (8.3%)	3 (23.1%)	1 (20%)	12 (6.7%)	2 (10.5%)
Ventricular morphology					
Left	132 (60.8%)	9 (69.2%)	4 (80%)	115 (63.9%)	4 (21.1%)
Right	65 (30%)	4 (30.8%)	1 (20%)	46 (25.6%)	14 (73.7%)
Biventricular/indeterminate	20 (9.2%)	0 (0%)	0 (0%)	19 (10.6%)	1 (5.3%)
Anatomical group					
Tricuspid atresia	54 (24.9%)	4 (30.8%)	0 (0%)	49 (27.2%)	1 (5.3%)
HLHS	21 (9.7%)	0 (0%)	0 (0%)	15 (8.3%)	6 (31.6%)
DORV	31 (14.3%)	3 (23.1%)	1 (20%)	23 (12.8%)	4 (21.1%)
CAVC	11 (5.1%)	0 (0%)	1 (20%)	7 (3.9%)	3 (15.8%)
CAVC-DORV	13 (6%)	2 (15.4%)	0 (0%)	9 (5%)	2 (10.5%)
TGA	12 (5.5%)	1 (7.7%)	1 (20%)	10 (5.6%)	0 (0%)
ccTGA	10 (4.6%)	1 (7.7%)	0 (0%)	8 (4.4%)	1 (5.3%)
DILV	37 (17.1%)	2 (15.4%)	1 (20%)	33 (18.3%)	1 (5.3%)
PA-IVS	20 (9.2%)	0 (0%)	1 (20%)	18 (10%)	1 (5.3%)
Other	8 (3.7%)	0 (0%)	0 (0%)	8 (4.4%)	0 (0%)
Pre-Fontan procedures					
No. of prior procedures	4 (3)	1 (0)	1 (0)	4 (3)	5 (2)
Endovascular	1 (3)	0 (0)	0 (0)	2 (2)	3 (2.5)
Open	2 (0)	1 (0)	1 (0)	2 (0)	2 (1)
Aortic arch intervention	48 (22.1%)	0 (0%)	0 (0%)	42 (23.3%)	6 (31.6%)
BT or central shunt	86 (39.6%)	8 (61.5%)	3 (60%)	67 (37.2%)	8 (42.1%)
Pulmonary artery banding	93 (42.9%)	3 (23.1%)	1 (20%)	80 (44.4%)	9 (47.4%)
Norwood	24 (11.1%)	0 (0%)	0 (0%)	20 (11.1%)	4 (21.1%)
BCPA	196 (90.3%)	1 (7.7%)	0 (0%)	176 (97.8%)	19 (100%)
Age at BCPA (months)	8.8 (8.7)	102.3 (0)		8.6 (8.3)	11.5 (12.3)
Fontan operative characteristics					
Type of TCPC					
Intracardiac	21 (9.7%)	13 (100%)	5 (100%)	2 (1.1%)	1 (5.3%)
Extracardiac	196 (90.3%)	0 (0%)	0 (0%)	178 (98.9%)	18 (94.7%)
Age at TCPC (years)	3.7 (1.6)	4.6 (5.1)	6.4 (4)	3.6 (1.5)	4.5 (3.8)
Conduit size (mm)	18 (2)	16 (2)	16 (0)	18 (2)	18 (2)
Fenestration	192 (88.5%)	2 (15.4%)	0 (0%)	171 (95%)	19 (100%)
Fenestration size (mm)	4.5 (0.3)	3.75 (1.25)		4.5 (0)	5 (0.75)
Postoperative data					
No. of late interventions	1 (1)	0 (1)	1 (1)	1 (1)	3 (4.5)
Endovascular	1 (1)	0 (1)	0 (1)	1 (1)	2 (4)
Open	0 (0)	0 (0)	0 (0)	0 (0)	0 (1)
Fenestration open at last follow-up	28 (12.9%)	1 (7.7%)	0 (0%)	21 (11.7%)	6 (31.6%)
Pacemaker at last follow-up	24 (11.1%)	2 (8.3%)	1 (20%)	14 (7.8%)	7 (36.8%)
Follow-up after Fontan (years)	12.7 (10.5)	30.5 (2.1)	31 (31.6)	12.3 (9.8)	16.5 (12.4)

Data are presented as median (interquartile range) or frequencies (percentages).

BCPA: bidirectional cavopulmonary anastomosis; BT: Blalock-Taussig; CAVC: complete atrioventricular canal; CAVC-DORV: complete atrioventricular canal with double outlet right ventricle; ccTGA: congenitally corrected TGA; DILV: double inlet left ventricle; DORV: double outlet right ventricle; GA: transposition of the great arteries; HLHS: hypoplastic left heart syndrome; PA-IVS: pulmonary atresia with intact ventricular septum; TCPC: total cavopulmonary connection.

### Fontan failure

**Figure 3:**
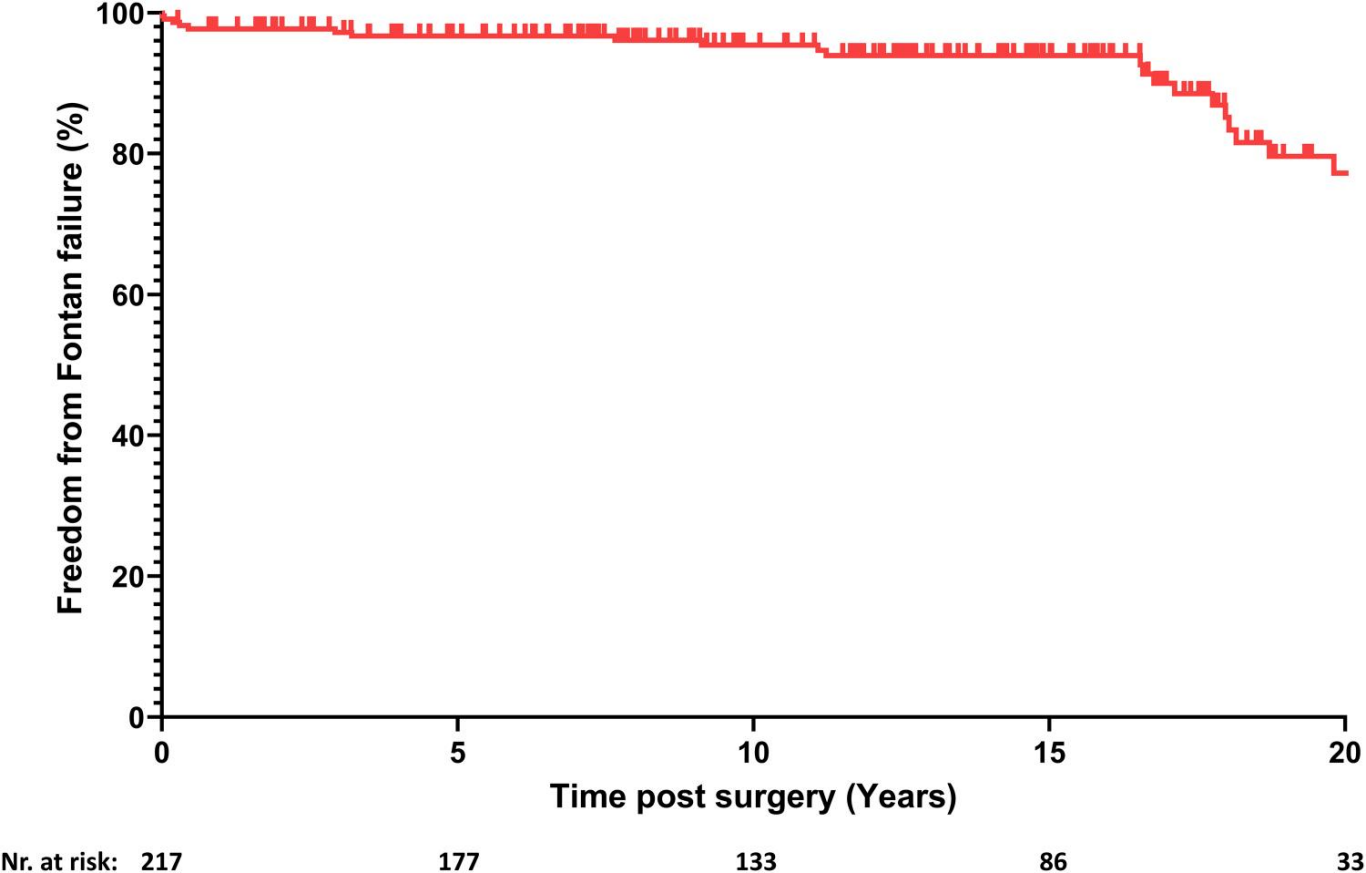
Kaplan–Meier function showing estimated freedom from Fontan failure.

A total of 24 patients (11.1%) experienced failure of the Fontan circulation (Table 2). The linearized risk of Fontan failure in our cohort is approximately 0.851% per year. The estimated freedom from Fontan circulatory failure was 97.7% (95% CI, 95–99) at 1 year and 77.2% (95% CI, 65–86) at 20 years of follow-up (Fig. 3). Seven patients (3.2%) died during the follow-up period. In Era I, 2 deaths occurred in the early postoperative period. Era II saw 5 late deaths, occurring after 2, 4 and 6 months, and after 3 and 18 years. Five patients (2.3%) underwent heart transplantation, one after 2 months and the others after 6, 11, 12 and 17 years. The estimated transplant-free survival was 98.1% (95% CI, 95–99) at 1 year and 87.2% (95% CI, 76–93) at 20 years of follow-up. Eight patients (3.7%) developed PLE after a median of 6.1 years (IQR, 3.3–15.4). One patient (0.5%) developed plastic bronchitis 14 years after Fontan completion.

**Table 2:** Morbidity and mortality after Fontan completion

	All Fontan patients (*n* = 217)	Era I (*n* = 18)	Era II (*n* = 199)
Fontan failure	24 (11.1%)	5 (27.8%)	19 (9.5%)
Death	7 (3.2%)	2 (11.1%)	5 (2.5%)
Early	2 (0.9%)	2 (11.1%)	0 (0%)
Late	5 (2.3%)	0 (0%)	5 (2.5%)
Heart transplantation	5 (2.3%)	0 (0%)	5 (2.5%)
Protein-losing enteropathy	8 (3.7%)	2 (11.1%)	6 (3%)
Plastic bronchitis	1 (0.5%)	0 (0%)	1 (0.5%)
NYHA III/IV	3 (1.4%)	1 (5.6%)	2 (1%)

Data are presented as frequencies (percentages).

NYHA: New York Heart Association Functional Classification.

### Causes of failure

The different causes of failure observed in the study are summarized in Table 3. The leading cause of failure was systolic ventricular dysfunction, which was responsible for 7 cases. This was trailed by AVV regurgitation, high PVR, and restrictive pathophysiology, each accounting for 4 cases, and obstruction, which accounted for 3 cases. In 2 patients with late Fontan failure, the specific cause of failure could not be determined because no catheterisation data were available. Patients with obstruction experienced early postoperative failure, due to thromboembolic events or critical stenosis, leading to mortality in the early postoperative period. AVV regurgitation was associated in all cases with complete atrioventricular canal defects and either biventricular morphology or RV morphology, with only one patient surviving at last follow-up. All patients with systolic ventricular dysfunction had RV dominance while most patients with restrictive pathophysiology had LV dominance. Additionally, all patients with restrictive pathophysiology developed PLE, and one patient required cardiac transplantation due to PLE.

**Table 3:** Patient characteristics according to the causes of failure

	Undetermined (*n* = 2)	Obstruction (*n* = 3)	AVV regurgitation (*n* = 4)	Systolic dysfunction (*n* = 7)	High PVR (*n* = 4)	Restrictive pathophysiology (*n* = 4)
Patient characteristics						
Left dominant morphology	1 (50%)	2 (66.7%)	0 (0%)	0 (0%)	2 (50%)	3 (75%)
Right dominant morphology	1 (50%)	1 (33.3%)	3 (75%)	7 (100%)	2 (50%)	1 (25%)
Age at Fontan completion (years)	7.44 (0.31)	3.79 (1.09)	10.62 (5.27)	3.65 (0.97)	5.21 (5.14)	4.87 (3.7)
Fenestration	1 (50%)	1 (33.3%)	4 (100%)	7 (100%)	4 (100%)	2 (50%)
Fenestration open at last follow-up	1 (50%)	1 (33.3%)	1 (25%)	3 (42.9%)	1 (25%)	0 (0%)
Time until Fontan failure (years)	16.2 (14.1)	0.04 (0.09)	9.96 (12.88)	3.34 (6.48)	2.33 (5.78)	8.57 (16.07)
Outcome						
Death/HTx	0 (0%)	3 (100%)	3 (75%)	4 (57.1%)	1 (25%)	1 (25%)
PLE/PB	0 (0%)	0 (0%)	1 (25%)	3 (42.9%)	2 (50%)	3 (75%)
NYHA III/IV	2 (100%)	0 (0%)	0 (0%)	0 (0%)	1 (25%)	0 (0%)

Data are presented as median (interquartile range) or frequencies (percentages).

AVV: atrioventricular valve; HTx: heart transplantation; NYHA: New York Heart Association Functional Classification; PB: plastic bronchitis; PLE: protein-losing enteropathy.

## DISCUSSION

Despite the reported favourable survival outcomes associated with TCPC, there remains a significant risk of late failure of the Fontan circulation. Consequently, there is also an anticipated rise in the number of Fontan patients experiencing circulatory failure [12]. The specific pathophysiological pathways leading to failure in TCPC patients are not yet fully understood. Understanding these mechanisms is crucial for guiding treatment strategies, facilitating personalized management and developing preventive measures.

Our study projects Fontan failure in approximately 10% of TCPC patients within 15 years postoperatively, and 25% beyond 25 years. A recent study involving 163 extracardiac conduit TCPC patients, reported 90% freedom from Fontan failure within 15 years postoperatively [13]. Contrastingly, the Australia and New Zealand Fontan Registry, which includes older surgical strategies, showed lower freedom from Fontan failure rates: 83% at 15 years and 56% at 25 years [2, 13].

When we categorized the patients based on the cause of failure, we observed that over 10% of them experienced early failure due to obstruction. Implementing early surgical or catheter intervention strategies to optimize the Fontan circulation and alleviate these obstructions could potentially prevent failure during the immediate postoperative period and enhance survival rates. In our study, we did not observe any instances of late Fontan failure in patients that could be directly linked to obstructions. This may be attributed to our institution's proactive approach towards managing any narrowing within the Fontan connections in symptomatic and asymptomatic patients [14].

Systolic ventricular dysfunction was the main cause of failure in the majority of failing Fontan patients (29%). This finding is consistent with a recent study that observed a significant decrease in ejection fraction among patients with Fontan failure compared to those without [15]. Furthermore, all failing Fontan patients with systolic ventricular dysfunction in our study had RV dominance. This is not surprising, as previous research has shown that patients with systemic RV are prone to systemic ventricular dysfunction [16]. The morphology of the dominant ventricle plays also a crucial role in the clinical outcomes in the late stages following Fontan palliation and is associated with a 2.38-fold greater risk factor for mortality compared to LV dominance [17]. Our study confirmed a higher incidence of RV dominance in patients with failing Fontan physiology compared to LV dominance. Moreover, in Fontan patients with RV dominance, we observed a gradual decline in systolic ventricular function, with at least mild impairment in 35% after 10 years and 48% after 15 years. The potential effectiveness of standard heart failure medication for delaying or preventing ventricular dysfunction and avoiding Fontan failure in patients with RV dominance remains uncertain [18, 19]. Following Fontan failure with systolic ventricular failure, mechanical circulatory support with a ventricular assist device [20] or heart transplantation [21] is crucial in reducing morbidity and mortality.

There is also a concerning trend of progressive decline in AVV function following the Fontan procedure, which may ultimately result in Fontan failure [15, 22]. Our study identified AVV regurgitation in 4 of the failing Fontan patients as the causes of failure. This finding contrasts with the results of a recent study by King *et al.*, who reported a higher incidence of Fontan failure with AVV failure (21.2%) in a cohort of 1199 patients. King *et al.*’s [23] study also highlighted the independent risk factor of RV dominance for AVV failure, which increased the risk of AVV failure by more than 2-fold. In our study, we have also observed that most failing Fontan patients with AVV regurgitation exhibited RV dominance. However, it is crucial to note that all patients in our study had complete atrioventricular canal defects, suggesting that the development of AVV failure in our patients is related to a morphological AVV dysfunction, rather than RV failure. The effectiveness of reoperation in preventing or reversing Fontan failure in this specific patient population is still unclear, given the challenges associated with valve repair or replacement. Menon *et al.* [24] reported that one-third of patients who underwent AVV surgery following Fontan palliation died within 5 years of the procedure.

High PVR has long been considered the critical bottleneck of the Fontan circulation, determining cardiac output by limiting preload reserve [25]. Recent research suggests that an increase in PVR is rarely observed and that is not the leading cause of Fontan failure [15]. Interestingly, our study also attributed Fontan failure due to high PVR to only 4 patients. These patients could potentially benefit from pulmonary vasodilators, although their effectiveness is often limited and varies greatly between patients [26]. Traditionally, elevated PVR was considered a contraindication to heart transplantation. However, emerging literature on PVR suggests that heart transplantation is also a viable option for patients with pulmonary hypertension [27]. Unlike patients with systolic ventricular dysfunction who require a ventricular assist device, these patients can potentially benefit from the implantation of a cavopulmonary assist device [28].

Our previous research demonstrated that restrictive pathophysiology can be a crucial cause of Fontan failure [7]. In the Fontan circulation, chronic preload deprivation of the systemic ventricle is associated with a decrease in ventricular compliance. This leads to poor ventricular filling and a progressive decline in cardiac output. Among adults with Fontan palliation and restrictive pathophysiology, an isolated elevation of ventricular end-diastolic pressure is often the only significant finding from cardiac catheterization [29]. We observed restrictive pathophysiology in approximately 20% of failing Fontan patients. Another recent study found that 31% of failing Fontan patients had diastolic dysfunction [15]. It is unclear if the restrictive pathophysiologic changes in these patients are reversible and if gradually increasing ventricular preload could restore restrictive pathophysiology. Preload optimization could be achieved through various mechanisms, such as pharmacologically reducing PVR, improving cardiovascular fitness, creating a fenestration to introduce a right-to-left shunt, or implanting a cavopulmonary assist device [30, 31].

The strengths of this study lie in its extensive scope, spanning over a 30-year period and encompassing a large group of patients. This long-term analysis provides a comprehensive understanding of the causes of Fontan failure. Furthermore, the collective discussion of all patients in the heart team and the consistency of this team over the 30-year duration ensures a unified approach to patient care and decision-making, adding to the robustness of the study.

### Limitations

Study limitations include its retrospective, single-institution design and long timeframe with evolving management practices. The influence of these factors on long-term outcomes and the prevalence of various causes of failure could not be evaluated in this retrospective study. Moreover, accounting for these factors would be challenging due to the heterogeneity of the study population. Furthermore, the qualitative assessment of ventricular function and AVV function through echocardiography has inherent limitations. The echocardiographic evaluation was conducted under resting conditions, which may not reflect ventricular dysfunction or AVV regurgitation that may manifest during exercise. The causes of failure were classified retrospectively based on clinical impressions. These predefined causes of failure were retrospectively determined from medical records and prioritized based on their assumed order of occurrence, potentially introducing misclassification bias. Additionally, our study could not directly assess ventricular relaxation and compliance, hence restrictive pathophysiology was defined by an unobstructed circulation, absence of AVV regurgitation, normal systolic function, and a low TPG with increased ventricular filling pressures. Consequently, it is plausible that our study underestimates the true extent to which restrictive pathophysiology contributes to Fontan failure.

## CONCLUSION

The findings of our study indicate that approximately 10% of patients who underwent TCPC Fontan palliation are projected to experience Fontan failure within a 15-year timeframe. Our research identified 5 distinct causes leading to failure and revealed that the group of failing Fontan patients is heterogeneous, with individuals distributed evenly among these causes. Among those with RV dominance, there was a progressive decline in ventricular function, with systolic dysfunction being the primary cause of late Fontan failure. Conversely, patients with LV dominance exhibited failure due to restrictive pathophysiology or high PVR. Moving forward, comprehensive multi-institutional studies are necessary to thoroughly characterize the patient characteristics associated with each cause of failure. This will facilitate the development of algorithms aimed at guiding personalized prophylactic or curative management strategies, thereby potentially delaying or preventing the adverse outcomes observed in Fontan patients.

## Data Availability

The datasets generated during and/or analysed during the current study are available from the corresponding author on reasonable request.

## ABBREVIATIONS

AVV: Atrioventricular valve
CI: Confidence interval
IQR: Interquartile range
LV: Left ventricular
NYHA: New York Heart Association Functional Classification
PLE: Protein-losing enteropathy
PVR: Pulmonary vascular resistance
RV: Right ventricular
TCPC: Total cavopulmonary connection
TPG: Transpulmonary gradient

## References

[1] Fontan F , Baudet E. Surgical repair of tricuspid atresia. Thorax 1971;26:240–8. 5089489 10.1136/thx.26.3.240 PMC1019078

[2] D’udekem Y , Iyengar AJ, Galati JC, Forsdick V, Weintraub RG, Wheaton GR et al Redefining expectations of long-term survival after the Fontan procedure twenty-five years of follow-up from the entire population of Australia and New Zealand. Circulation 2014;130:S32–8. 25200053 10.1161/CIRCULATIONAHA.113.007764

[3] Kverneland LS , Kramer P, Ovroutski S. Five decades of the Fontan operation: a systematic review of international reports on outcomes after univentricular palliation. Congenit Heart Dis 2018;13:181–93. 29372588 10.1111/chd.12570

[4] Dennis M , Zannino D, Du Plessis K, Bullock A, Disney PJS, Radford DJ et al Clinical Outcomes in Adolescents and Adults After the Fontan Procedure. J Am Coll Cardiol 2018;71:1009–17. 29495980 10.1016/j.jacc.2017.12.054

[5] John AS. Fontan repair of single ventricle physiology. Consequences of a unique physiology and possible treatment options. Cardiol Clin 2015;33:559–69, viii. 26471820 10.1016/j.ccl.2015.07.010

[6] Murtuza B , Hermuzi A, Crossland DS, Parry G, Lord S, Hudson M et al Impact of mode of failure and end-organ dysfunction on the survival of adult Fontan patients undergoing cardiac transplantation. Eur J Cardiothorac Surg 2017;51:135–41. 27401703 10.1093/ejcts/ezw243

[7] Van Puyvelde J , Verbeken E, Gewillig M, Meyns B. Fontan failure associated with a restrictive systemic ventricle. J Thorac Cardiovasc Surg 2017;154:e7–8. 28292590 10.1016/j.jtcvs.2017.02.016

[8] Backer CL , Russell HM, Pahl E, Mongé MC, Gambetta K, Kindel SJ et al Heart transplantation for the failing Fontan. Ann Thorac Surg 2013;96:1413–9. 23987899 10.1016/j.athoracsur.2013.05.087

[9] Griffiths ER , Kaza AK, Wyler von Ballmoos MC, Loyola H, Valente AM, Blume ED et al Evaluating failing Fontans for heart transplantation: predictors of death. Ann Thorac Surg 2009;88:558–63. 19632412 10.1016/j.athoracsur.2009.03.085 PMC2844259

[10] Simpson KE , Cibulka N, Lee CK, Huddleston CH, Canter CE. Failed Fontan heart transplant candidates with preserved vs impaired ventricular ejection: 2 distinct patient populations. J Heart Lung Transplant 2012;31:545–7. 22381208 10.1016/j.healun.2012.02.003

[11] Hansmann G , Koestenberger M, Alastalo TP, Apitz C, Austin ED, Bonnet D et al 2019 updated consensus statement on the diagnosis and treatment of pediatric pulmonary hypertension: the European Pediatric Pulmonary Vascular Disease Network (EPPVDN), endorsed by AEPC, ESPR and ISHLT. J Heart Lung Transplant 2019;38:879–901. 31495407 10.1016/j.healun.2019.06.022

[12] Schilling C , Dalziel K, Nunn R, Du Plessis K, Shi WY, Celermajer D et al The Fontan epidemic: population projections from the Australia and New Zealand Fontan Registry. Int J Cardiol 2016;219:14–9. 27257850 10.1016/j.ijcard.2016.05.035

[13] Rijnberg FM , Blom NA, Sojak V, Bruggemans EF, Kuipers IM, Rammeloo LAJ et al A 45-year experience with the Fontan procedure: tachyarrhythmia, an important sign for adverse outcome. Interact CardioVasc Thorac Surg 2019;29:461–8. 31038168 10.1093/icvts/ivz111

[14] Salaets T , Cools B, De Meester P, Heying R, Boshoff D, Eyskens B et al Stent expansion of restrictive Fontan conduits to nominal diameter and beyond. Catheter Cardiovasc Interv 2022;100:1059–66. 36321584 10.1002/ccd.30438

[15] Sallmon H , Ovroutski S, Schleiger A, Photiadis J, Weber SC, Nordmeyer J et al Late Fontan failure in adult patients is predominantly associated with deteriorating ventricular function. Int J Cardiol 2021;344:87–94. 34563595 10.1016/j.ijcard.2021.09.042

[16] Ansari Ramandi MM , Yarmohammadi H, Gareb B, Voors AA, van Melle JP. Long-term outcome of patients with transposition of the great arteries and a systemic right ventricle: a systematic review and meta-analysis. Int J Cardiol 2023;389:131159. 37433408 10.1016/j.ijcard.2023.131159

[17] Ponzoni M , Azzolina D, Vedovelli L, Gregori D, Di Salvo G, D’Udekem Y et al Ventricular morphology of single-ventricle hearts has a significant impact on outcomes after Fontan palliation: a meta-analysis. Eur J Cardiothorac Surg 2022;62:ezac535. 36367236 10.1093/ejcts/ezac535

[18] Budts W , Roos-Hesselink J, Rädle-Hurst T, Eicken A, McDonagh TA, Lambrinou E et al Treatment of heart failure in adult congenital heart disease: a position paper of the Working Group of Grown-Up Congenital Heart Disease and the Heart Failure Association of the European Society of Cardiology. Eur Heart J 2016;37:1419–27. 26787434 10.1093/eurheartj/ehv741 PMC4914888

[19] Baumgartner H , de Backer J, Babu-Narayan SV, Budts W, Chessa M, Diller GP, ESC Scientific Document Group et al 2020 ESC Guidelines for the management of adult congenital heart disease. Eur Heart J 2021;42:563–645. 32860028 10.1093/eurheartj/ehaa554

[20] Bedzra EKS , Adachi I, Peng DM, Amdani S, Jacobs JP, Koehl D et al; Society of Thoracic Surgeons Fontan VAD Group. Systemic ventricular assist device support of the Fontan circulation yields promising outcomes: an analysis of The Society of Thoracic Surgeons Pedimacs and Intermacs Databases. J Thorac Cardiovasc Surg 2022;164:353–64. 35016782 10.1016/j.jtcvs.2021.11.054

[21] Simpson KE , Pruitt E, Kirklin JK, Naftel DC, Singh RK, Edens RE et al Fontan patient survival after pediatric heart transplantation has improved in the current era. Ann Thorac Surg 2017;103:1315–20. 27863728 10.1016/j.athoracsur.2016.08.110

[22] Bove T , Grootjans E, Naessens R, Martens T, De Wolf D, Vandekerckhove K et al Long-term follow-up of atrioventricular valve function in Fontan patients: effect of atrioventricular valve surgery. Eur J Cardiothorac Surg 2023;64:ezad305. 37682065 10.1093/ejcts/ezad305

[23] King G , Ayer J, Celermajer D, Zentner D, Justo R, Disney P et al Atrioventricular valve failure in Fontan palliation. J Am Coll Cardiol 2019;73:810–22. 30784675 10.1016/j.jacc.2018.12.025

[24] Menon SC , Dearani JA, Cetta F. Long-term outcome after atrioventricular valve surgery following modified Fontan operation. Cardiol Young 2011;21:83–8. 20977827 10.1017/S1047951110001538

[25] Gewillig M , Brown SC. The Fontan circulation after 45 years: update in physiology. Heart 2016;102:1081–6. 27220691 10.1136/heartjnl-2015-307467 PMC4941188

[26] Goldberg DJ , Zak V, Goldstein BH, Schumacher KR, Rhodes J, Penny DJ et al; Pediatric Heart Network Investigators. Results of the FUEL trial. Circulation 2020;141:641–51. 31736357 10.1161/CIRCULATIONAHA.119.044352 PMC7042084

[27] Kaza AK , Kaza E, Bullock E, Reyna S, Yetman A, Everitt MD. Pulmonary vascular remodelling after heart transplantation in patients with cavopulmonary connection. Eur J Cardiothorac Surg 2015;47:505–10; discussion 510. 24819364 10.1093/ejcts/ezu198

[28] Rychik J , Atz AM, Celermajer DS, Deal BJ, Gatzoulis MA, Gewillig MH et al; American Heart Association Council on Cardiovascular Disease in the Young and Council on Cardiovascular and Stroke Nursing. Evaluation and management of the child and adult with Fontan circulation: a scientific statement from the American Heart Association. Circulation 2019;140:e234-84–e284. 31256636 10.1161/CIR.0000000000000696

[29] Averin K , Hirsch R, Seckeler MD, Whiteside W, Beekman RH, Goldstein BH. Diagnosis of occult diastolic dysfunction late after the Fontan procedure using a rapid volume expansion technique. Heart 2016;102:1109–14. 26917538 10.1136/heartjnl-2015-309042

[30] d’Udekem Y , Van Puyvelde J, Rega F, Nix C, Barth S, Meyns B. Validating the concept of mechanical circulatory support with a rotary blood pump in the inferior vena cava in an ovine Fontan model. Bioengineering 2024;11:594. 38927830 10.3390/bioengineering11060594 PMC11200902

[31] Budts W , Ravekes WJ, Danford DA, Kutty S. Diastolic heart failure in patients with the Fontan circulation. JAMA Cardiol 2020;5:590–7. 32022823 10.1001/jamacardio.2019.5459

